# A Preferable Approach for the Quality Control of Xiaoer Chiqiao Qingre Granules Based on the Combination of Chromatographic Fingerprints and Chemometrics

**DOI:** 10.1155/2020/6836981

**Published:** 2020-09-29

**Authors:** Tong Xu, Xiaoqi Li, Mengmeng Huang, Qi Wang, Chao Li, Gang Tian, Yan Chen

**Affiliations:** ^1^Affiliated Hospital of Integrated Traditional Chinese and Western Medicine, Nanjing University of Chinese Medicine, Nanjing 210028, China; ^2^The State Key Laboratory of Natural Medicines, China Pharmaceutical University, Nanjing 210009, China; ^3^Multi-Component of Traditional Chinese Medicine and Microecology Research Center, Jiangsu Province Academy of Traditional Chinese Medicine, Nanjing 210028, China; ^4^Jumpcan Pharmaceutical Co., Ltd., Taixing 225400, China

## Abstract

A preferable approach of a combination of a multiwavelength fusion HPLC fingerprint and chemometrics for the quality control of Xiaoer Chiqiao Qingre granules (XCQG) was established in this study. A single-wavelength HPLC fingerprint was performed to identify 18 peaks as common peaks in the beginning, and 12 of them were recognized by HPLC-Q/TOF-MS. To overcome the limitation of the single-wavelength HPLC fingerprint, a three-wavelength (230 nm, 250 nm, and 330 nm) fusion fingerprint was established for a more thorough quality assessment. Six main active ingredients (geniposide, paeoniflorin, forsythin, forsythoside A, baicalin, and wogonoside) were selected as chemical markers for simultaneous quantitative analysis, while the results indicated that the content of other five ingredients except forsythoside A presented comparatively stable. Chemometrics including hierarchical cluster analysis (HCA) and orthogonal partial least squares discriminant analysis (OPLS-DA) were performed to evaluate the homogeneity and heterogeneity of sixteen batches of XCQG. The results of the multiwavelength fingerprint were clearly classified into two clusters by HCA, whereas the single-wavelength fingerprint showed no distinct difference between them. OPLS-DA was further employed to prove that the above six main active ingredients made great contributions to clustering. In summary, this integrated analysis provided a better promoted and more comprehensive method to control the quality of XCQG.

## 1. Introduction

Xiaoer Chiqiao Qingre granules (XCQG) are a traditional Chinese medicine (TCM) preparation widely used in the clinical treatment of children's flu. It consists of fourteen Chinese herbs involving *Forsythiae Fructus* (FF), *Sojae Semen Praeparatum* (SSP), *Menthae Haplocalycis Herba* (MHH), *Schizonepetae Herba* (SH), *Gardeniae Fructus* (GF), *Rhei Radix Et Rhizoma* (RRER), *Artemisiae annuae Herba* (AAH), *Paeoniae Radix Rubra* (PRR), *Arecae Semen* (AS), *Magnoliae officinalis cortex* (MOC), *Scutellariae Radix* (SR), *Pinelliae Rhizoma* (PR), *Bupleuri Radix* (BR), and *Glycyrrhizae Radix Et Rhizoma* (GRER). XCQG possesses the function of dispelling wind, relieving the exterior, clearing heat, and removing food stagnation. Several pharmacological studies have demonstrated that XCQG has a good effect on anti-inflammation, antibacteria, and antivirus [1–6], as well as the treatment of children's viral fever, headache, and tonsillitis.

For its remarkable clinical effects, XCQG is recorded in China Pharmacopeia 2015, and geniposide from GF and forsythin from FF are selected as two indicators for the quality control of XCQG. However, many chemical components in XCQG have been reported as active compounds and have pharmacological actions. For example, paeoniflorin from PRR possesses obvious antioxidative and anti-inflammatory properties [2, 3], and forsythoside A from FF also has remarkable antibacterial and antiviral effects [4, 5]. In addition, baicalin and wogonoside as abundant flavonoid glycosides from SR also exhibit obvious anti-inflammatory property [3, 6]. Therefore, in order to better control the quality of XCQG, multiple active components containing geniposide, paeoniflorin, forsythin, forsythoside A, baicalin, and wogonoside should be chosen as new activity markers to evaluate the quality of XCQG.

Chromatographic fingerprint is a comprehensive, quantifiable analysis method and nowadays is widely used to evaluate the quality and consistency of complicated TCM [7, 8]. It can characterize both known and unknown components in a complex mixture and present in the form of a chromatogram [9]. Currently, it has become a powerful and practical tool for the comprehensive analysis of multicomponents in TCM or their preparations [10–13]. Due to the complexity of TCM preparations, the single-wavelength fingerprint can only collect part of the chromatographic information of complex compounds. It is difficult to fully represent the overall information in the preparation if it is only based on the current standard or analytical methods. Therefore, in order to avoid the limitation caused by single-wavelength determination, a multiwavelength fusion fingerprint analysis method [10, 14] is established in this paper. The HPLC data at different wavelengths are fused into one chromatogram, which fully reflects the comprehensive information in the sample. Additionally, it is also a common practice to combine fingerprint with chemometrics [15, 16], such as HCA and OPLS-DA, which could maximize the inherent information obtained from the fingerprint [17–20]. The combination of multiwavelength fingerprint and chemometrics could be a promising tool for comprehensive and systematic analysis of TCM to better control the quality of complex TCM preparation.

In this study, a three-wavelength fused fingerprint (230 nm, 250 nm, and 330 nm) of 16 batches of XCQG was established, and the contents of six markers including geniposide, paeoniflorin, forsythin, forsythoside A, baicalin, and wogonoside were simultaneously determined. Meanwhile, HCA and OPLS-DA were employed to assess the resemblance and dissimilarities of different samples and to obtain variables contributing most to the difference. This comprehensive method provides a significant reference for the quality control of XCQG [21, 22].

## 2. Experimental

### 2.1. Materials and Reagents

Sixteen batches of XCQG samples (nos. 1609164, 1610214, 1611284, 1612034, 1612354, 1612454, 1612484, 1701024, 1702134, 1702264, 1705114, 1705124, 1705134, 1705144, 1705154, and 1705164) were collected from Jumpcan Pharmaceutical Co., Ltd. (Taizhou, China) for this study. Geniposide (purity 97.6%, LOT 110749–201718), paeoniflorin (purity 95.7%, LOT 110736–201741), forsythoside A (purity 93.4%, LOT 111810–201606), forsythin (purity 94.9%, LOT 110821–201615), and wogonoside (purity 98.5%, LOT 112002–201702) were purchased from National Institute for the Control of Pharmaceutical and Biological Products (China), and baicalin (purity ≥ 98%, LOT P16S8F44143) was provided by Shanghai Yuanye Biotechnology Co., Ltd. (Shanghai, China). Acetonitrile (HPLC grade, TEDIA^®^) was obtained from Nanjing Liangwei Biotechnology Co., Ltd. (Nanjing, China). Water was purified by a Millipore Elix^®^ Essential 5 system (USA). All other chemicals and solvents were of analytical grade.

### 2.2. Preparation of Standard Solutions

Stock solution was a mixture of 253.0 *μ*g/mL of geniposide, 139.7 *μ*g/mL of paeoniflorin, 450.2 *μ*g/mL of forsythoside A, 593.3 *μ*g/mL of baicalin, 126.9 *μ*g/mL of forsythin, and 220.0 *μ*g/mL of wogonoside, which was dissolved in 70% methanol. A series of mixed standard solutions were prepared by diluting the stock solution with 70% methanol. All the standard solutions were passed through a suitable filter of 0.45 *μ*m pore size before injected into the HPLC instrument.

### 2.3. Preparation of Sample Solutions

For the chromatography fingerprint analysis, 1 g of the XCQG sample was accurately weighed and extracted with 20 mL methanol by sonication for 30 min (250 W, 45 kHz). An appropriate amount of methanol was added to the cooled extract to make up the lost weight. Then, the supernatant was filtrated through a 0.22 *μ*m membrane filter and injected into the HPLC instrument for analysis.

For the quantitative analysis, an accurately weighed XCQG sample of 0.3 g was extracted with 25 mL of 70% methanol by sonication for 30 min (250 W, 45 kHz).The extract cooled down at room temperature, and then 70% methanol was added to the extract to compensate the weight loss of the solvent. The supernatant was filtrated by a 0.22 *μ*m membrane filter and injected into the HPLC instrument for analysis.

### 2.4. Apparatus and Chromatographic Conditions

All the HPLC analyses were performed using an Agilent 1260 HPLC system equipped with a quaternary pump, an autosampler, a diode array detector, and a thermostatically controlled column compartment. Separations were conducted on an Agilent Zorbax SB-C_18_ column (250 × 4.6 mm, 5 *μ*m).

#### 2.4.1. Conditions of HPLC Fingerprint Analysis

For the HPLC fingerprint analysis, the mobile phase was a gradient of A (methanol : acetonitrile = 2 : 1) and B (0.1% formic acid/H_2_O): 12% A for 0–10 min; 12%–30% A for 10–30 min; 30%–33% A for 30–50 min; 33%–45% A for 50–75 min; 45%–96% A for 75–110 min; and 96% A for 110–120 min. The flow rate was set at 1.0 mL/min, the column temperature was maintained at 25°C, and the injection volume of the test sample was 5 *μ*L. The UV spectra were recorded at 230 nm, 250 nm, and 330 nm.

#### 2.4.2. Conditions of HPLC Quantitation

The gradient elution time and proportional composition of the mobile phase (A (acetonitrile) and B (0.1% phosphoric acid/H_2_O)) at the stated time were listed as follows: 5%–10% A for 0–10 min, 10%–13 % A for 10–18 min, 13% A for 18–33 min, 13%–16% A for 33–38 min, 16% A for 38–50 min, 16%–22% A for 50–55 min, 22% A for 55–65 min, 22%–32% A for 65–85 min, 32%–90% A for 85–86 min, and 90% A for 86–95 min. The flow rate was 1.0 mL/min, and the column temperature was kept at 30°C. The loading volume was 10 *μ*L, and the wavelength of UV spectra was 250 nm.

#### 2.4.3. HPLC-Q/TOF-MS Analysis Condition

The HPLC system was interfaced to Agilent 6538 Accurate-Mass Q-TOF LC/MS (USA), which was equipped with a DAD and an ESI source. The sample was analyzed in the negative mode, and the parameters were set as follows. The mass detector recorded a range between mass-ion ratios (m/z) 100 and 1000, the drying gas (N_2_) flow rate was 10 L/min, the drying gas temperature was 350°C, the nebulizer pressure was 40 psi, the capillary voltage was 3500 V, the skimmer was 65 V, and the fragment or voltage was 135 V. The data were processed using Agilent MassHunter Qualitative Analysis software (version B.03.01, Agilent Technologies). The other chromatographic conditions were as the same as the HPLC fingerprint conditions.

### 2.5. Data Analysis

HCA was employed using SPSS (19.0), and OPLS-DA was employed using SIMCA-P (13.0.3). The principal component analysis and other related programs were processed by the Matlab R2016b developed program. The retention time and peak areas of the fusion fingerprint were acquired from the Matlab R2016b developed program.

## 3. Results and Discussion

### 3.1. Optimization of Sample Extract Conditions

In order to develop a viable and appropriate sample extract method for HPLC fingerprint analysis and quantitative analysis, a series of extract conditions were investigated in the article.

In the study of extract conditions for HPLC fingerprint analysis, different extract solvents (methanol, 70% methanol, and 70% ethanol) and different extract methods (sonication and reflux) were optimized [23–25]. The results showed that methanol was the best solvent, and sonication was the most appropriate method for the extract.

However, in the study of quantitative analysis, extract solvents (50% methanol, 70% methanol, and methanol), extract methods (sonication, reflux, and cold soak), extract volumes (25 mL, 50 mL, and 75 mL), and extract durations (30 min, 60 min, and 120 min) were investigated. The results showed that 25 mL 70% methanol sonication for 30 min was the most effective extract procedure for the sample analysis.

### 3.2. Optimization of Chromatographic Conditions

In order to obtain the maximum chemical information and the best separation of the fingerprint, different detection wavelengths and compositions of the mobile phase were optimized. Due to the complex composition of the mixture, the detection wavelength was scanned in the entire UV range (210 nm–400 nm). When the detection wavelength was set at 250 nm, most analytes showed a good response with little interference. At the same time, different mobile phases such as acetonitrile/phosphoric acid/H_2_O and methanol/acetonitrile/formic acid/H_2_O were examined, and the results demonstrated that the latter could obtain the better chromatographic performance.

Besides, for the quantitative study, to obtain the best separation of the 6 ingredients (geniposide, paeoniflorin, forsythin, forsythoside A, baicalin, and wogonoside), different column temperatures (25°C, 30°C, and 35°C), flow rates (0.8 mL/min, 1.0 mL/min, and 1.2 mL/min), and injection volumes (5 *μ*L, 10 *μ*L, and 15 *μ*L) were further optimized. It could be clearly obtained from the results that the resolutions and peak shapes of the investigated compounds were fairly good when the column temperature was kept at 30°C, the flow rate was set at 1.0 mL/min, and the injection volume was set at 10 *μ*L.

### 3.3. Method Validation

#### 3.3.1. Method Validation of the HPLC Fingerprint

In order to verify the reliability of the method, a randomly selected sample was chosen for the method validation. The precision, stability, and repeatability were assessed by calculating the similarity of test samples. The precision was assessed by continuously injecting the same sample 6 times under the above chromatographic conditions. The stability was assessed by analyzing the sample on, respectively, 0, 3, 6, 9, 12, 24, and 48 h, and the repeatability was evaluated by analyzing six samples which were prepared in parallel. The chromatograms were recorded, and the similarities were calculated by the “Similarity Evaluation System for Chromatographic Fingerprint of TCM” (2004A). The similarity of precision, stability, and repeatability was all above 0.95, indicating this method was applicable, reliable, and repeatable.

#### 3.3.2. Method Validation of HPLC Quantitative Determination

The optimized quantitative method was validated by assessing the linearity, precision, stability, repeatability, and recovery. The linearity was tested by measuring the above mixed standard solutions and recording the peak areas. Taking the peak areas as the *Y*-axis and the concentrations as the *X*-axis, the regression equations as well as the linear ranges were investigated. Precision was performed by continuously analyzing the sample six times, and the RSDs of the peak areas of the above six ingredients were found in the range of 0.41%–2.34%. Stability was assessed by testing the same sample at different times (0, 2, 4, 8, 12, and 24 h), and the RSDs of the peak areas were between 0.49% and 3.54%. To confirm the repeatability, three high-level, three medium-level, and three low-level samples were weighed in parallel, which, namely, were 0.45 g, 0.3 g, and 0.15 g, respectively. These nine sample solutions were prepared, and the contents of 6 ingredients were measured, while the RSDs of contents were 0.91%–2.16%. Then, the recovery study was measured by analyzing the spiked samples. 0.15 g of the sample was precisely weighed nine times with three different levels (0.5, 1.0, and 1.5 fold) of six reference compounds added to each sample, and the contents of six ingredients were calculated. The recovery was calculated by the formula recovery (%) = (amount found − original amount)/amount spiked × 100%. The recoveries of six ingredients ranged from 94.93% to 101.41%, and the RSDs ranged from 2.08% to 2.99%. These above results are all summarized in Table 1, showing the method was accurate, steady, and viable.

### 3.4. Sample Analysis

#### 3.4.1. Establishment of HPLC Fingerprint Chromatographic Conditions

**Figure 1:**
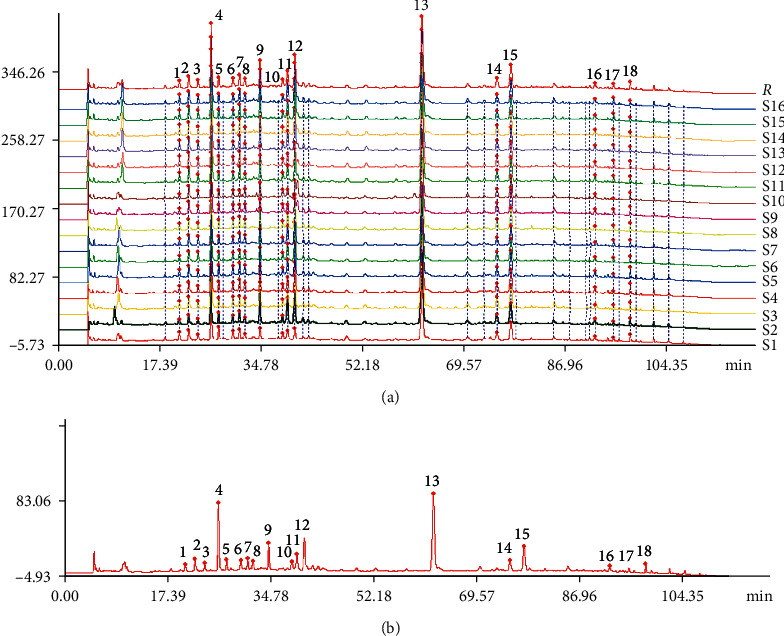
(a) HPLC fingerprint of XCQG samples. (b) Reference HPLC fingerprint of XCQG.

According to the chromatographic conditions under 2.4.1, sixteen batches of samples were tested. The corresponding recorded chromatograms were then imported to “Similarity Evaluation System of Chromatographic Fingerprint of TCM” (2004A) and further were matched through the method of multipoint correction. The HPLC fingerprint of XCQG samples (Figure 1(a)) and the reference HPLC fingerprint of XCQG (Figure 1(b)) were established. Eighteen peaks were selected as common peaks due to their good resolution and high response. The correlation coefficients between samples (from S1 to S16) and the reference fingerprint were 0.969, 0.982, 0.993, 0.982, 0.974, 0.986, 0.963, 0.988, 0.974, 0.970, 0.977, 0.979, 0.972, 0.970, 0.965, and 0.974, respectively. These results showed that XCQG from different batches were of a good consistency.

#### 3.4.2. Identification of Common Peaks by HPLC-Q/TOF-MS

**Figure 2:**
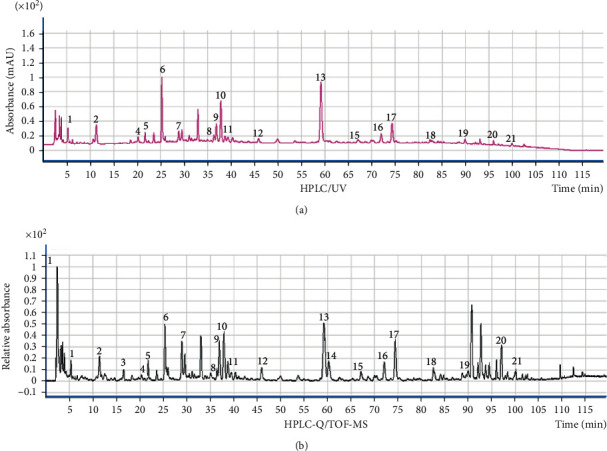
TIC chromatogram in the negative mode of XCQG: (a) HPLC/UV (250 nm); (b) HPLC-Q/TOF-MS.

The total ion current (TIC) chromatogram of the sample is shown in Figures 2(a) and 2(b). The identification results of eighteen common markers from the HPLC fingerprint of XCQG by HPLC-Q/TOF-MS, assigned by their retention time, MS and MS [2] spectral data with those of standard available compounds and previous publications, are summarized in Table 2. Retention time, order of elution, and mass-to-charge ratio were explicitly provided in the table. Twelve of these eighteen peaks were identified or preliminarily identified. Then, by comparison with reference substances, nine peaks were exactly identified, and the other three were presumed (nos. 10, 11, and 14) [26–28], including flavonoids (nos. 10, 13, 14, 15, and 16, from SR), phenylpropanoid glycosides (nos. 11 and 12, from FF), iridoids (nos. 2 and 4, from GF), organic acids (no. 1, from GF), triterpenoid saponin (no. 18, from GRER), and monoterpene glycoside (no.6, from PRR). Their structures are displayed in Figure S1.

#### 3.4.3. Quantitative Determination of the Six Ingredients

In this study, the contents of six ingredients in sixteen batches of samples were determined under the conditions specified in Section 2.4.2 by the external reference method. The methodological investigation presented satisfied results, shown in Table 1, which manifested that the HPLC method was completely feasible for the quantification. The contents of six ingredients are exhibited in Table 3, with their respective ranges obtained as follows: 4.49–5.42 mg·g^−1^ for geniposide, 2.55–3.98 mg·g^−1^ for paeoniflorin, 0.57–1.27 mg·g^−1^ for forsythin, 1.94–6.75 mg·g^−1^ for forsythoside A, and 7.75–11.37 mg·g^−1^ for baicalin. It could be clearly observed that the contents of six ingredients in different samples all met the criterion of Chinese Pharmacopoeia 2015 and were generally stable except for forsythoside A. As a main active ingredient of FF, forsythoside A had strong antibiotic and antiviral effects consistent with the functions and indications of XCQG, which enabled it to be an index component for the quantification of XCQG. However, compared with the other five ingredients, the contents of forsythoside A in sixteen batches presented a larger fluctuation, which was probably because the origins, used parts, and harvest time of FF of the sixteen batches were different. This distinct difference of forsythoside A reminded us to further strengthen the quality control of FF to ensure the quality and stability, as well as the clinical safety and effectivity of the preparation.

#### 3.4.4. Chemometric Analysis

**Figure 3:**
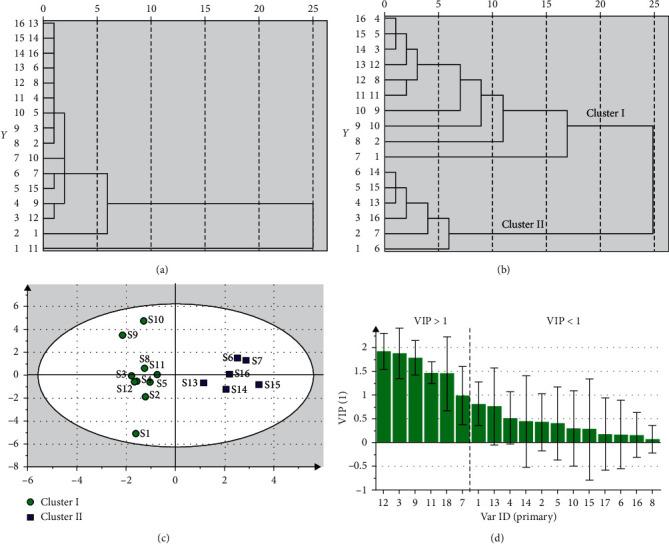
(a) Dendrogram of cluster analysis of 16 batches based on the single-wavelength fingerprint (250 nm). (b) Dendrogram of cluster analysis of 16 batches based on the multiwavelength fingerprint. (c) OPLS-DA score scatter plots of 16 batches of XCQG. (d) Variable important plot result.

The common peak areas of the single-wavelength fingerprints (*λ* = 250 nm) in different collected samples were transferred to SPSS 19.0, and HCA was performed. As can be seen from Figure 3(a), when the Euclidean distance was set at 10, all the samples were observed in one cluster except S11, which indicated that, except S11, the remaining samples were relatively steady.

Since single wavelength could only provide partial information of the complex compound groups, multiwavelength fusion technology was applied to obtain more information of ingredients, and the difference between two methods was further investigated in this paper. Based on the above strategy, three-wavelength fingerprints (230, 250, and 330 nm) of every sample were fused, and then the retention times and peak areas were calculated by self-programmed software. In order to evaluate the variety of these samples, HCA was employed again by using the calculated common peak areas as input data. As shown in Figure 3(b), the samples were classified into two clusters (cluster0049;and cluster 00490049), which indicated that there was a significant distinction between these samples. Meanwhile, to further investigate the difference of these two clusters, the OPLS-DA model was established after the samples were divided into two groups in advance according to the aforementioned result. It was indicated that the prediction ability of the model was fairly good as shown in Figure 3(c), with the regression model which possessed an explained variance (*R*^2^*Y*) of 0.925, principal component regression coefficient (*Q*^2^*Y*) of 0.719, and matrix setting rate of 0.589. Then, in order to further identify the components with great contribution to the sample classification, the components with great differences were screened by variable importance projection (VIP) in OPLS-DA, and the results are shown in Figure 3(d). Variables with VIP > 1.0 were selected as meaningful varieties, and a total of six varieties were chosen, which were peak 3, peak 7, peak 9, peak 11, peak 12 (forsythoside A), and peak 18. This result was consistent with the previous quantitative result, suggesting that forsythoside A might have a great contribution to the classification. In the meanwhile, it was confirmed that the strategy of fusion fingerprint was feasible and reliable and could provide more chemical information of the complex system.

## 4. Conclusion

In the study, single-wavelength and multiwavelength HPLC fingerprints were established, and eighteen chromatographic peaks selected as common peaks were further identified by HPLC-Q/TOF-MS. Six active markers of the sample in sixteen batches were simultaneously quantified, while forsythoside A varied significantly from different samples. HCA was then performed to investigate the resemblance and dissimilarities among the different samples. The results demonstrated that sixteen batches of XCQG samples could be clearly divided into two clusters based on the multiwavelength HPLC fingerprint, while they could not be represented in the result of single-wavelength HPLC fingerprint analysis. OPLS-DA was further employed to explore the differences between the two clusters. Six variables played dominating roles in clustering. Consistent with the quantitative results, forsythoside A had the largest VIP value, more probably satisfied to explain the classification. In conclusion, the new established fusion chromatography method combined with chemometric analysis was proved to be a more variable and reasonable method for the quality control of XCQG. This comprehensive method also offered a reference for the quality control of complex-compound Chinese herbal preparations.

## Figures and Tables

**Table 1:** Calibration curve, precision, stability, repeatability, and recovery values of six markers.

No.	Compound	Calibration curves	*R*	Linear range (*μ*g·mL^−1^)	Precision (RSD (%))	Repeatability (RSD (%))	Stability (RSD (%))	Recovery	
								Mean (%)	RSD (%)
1	Geniposide	*Y* = 12.22*X* + 19.55	0.9997	2.5–253.0	0.67	2.18	0.74	100.68	2.08
2	Paeoniflorin	*Y* = 13.52*X* – 1.84	0.9997	1.4–139.7	2.34	1.84	3.54	98.54	2.99
3	Forsythoside A	*Y* = 11.84*X* + 7.51	0.9995	4.5–450.2	0.41	0.91	0.53	97.26	2.54
4	Baicalin	*Y* = 15.54*X* + 49.18	0.9998	5.9–593.3	0.30	2.08	0.49	101.34	2.49
5	Forsythin	*Y* = 19.93*X* + 12.11	0.9998	1.3–126.9	0.78	1.43	0.79	94.93	2.87
6	Wogonoside	*Y* = 14.57*X* + 13.63	0.9998	2.2–220.0	0.67	2.16	0.88	101.41	2.43

**Table 2:** Identification of 18 common markers from the HPLC fingerprint of XCQG by HPLC-Q/TOF-MS.

No.	*t*_R_ (min)	Molecular weight (Da)	MS/MS (m/z)	Formula	Identification	Error (ppm)
1	20.310	354	353.0900[M-H]^−^, 191.0549[M-H-CA]^−^	C_16_H_18_O_9_	Chlorogenic acid	−5.17
2	21.723	550	595.1924[M-H]^−^, 549.1860[M-H]^−^, 225.0785[M-H-2Glc]^−^	C_23_H_34_O_15_	Genipin 1-gentiobioside	6.19
3	23.637		Unknown			
4	25.371	388	433.1416[M + HCOO]^−^, 225.0766[387-Glc]^−^, 207.0662[225-H_2_O]^−^, 123.0447[225-C_4_H_6_O_3_]^−^	C_17_H_24_O_10_	Geniposide	2.83
5	26.128		Unknown			
6	29.019	480	525.1655[M + HCOO]^−^, 327.1100	C_23_H_28_O_11_	Paeoniflorin	8.45
7	29.638	624	Unknown			
9	32.988	624	Unknown			
10	36.429	548	547.1490[M-H]^−^, 487.1276[M-H-60]^−^, 457.1168[M-H-90]^−^, 427.1068[M-H-120]^−^	C_26_H_28_O_13_	Chrysin 6-C-glucoside 8-C-arabinoside [26]	5.3
11	36.934	624	623.1994[M-H]^−^, 461.1685[M-CA]^−^	C_29_H_36_O_15_	Forsythoside I [27]	8.05
12	37.990	624	623.2052[M-H]^−^, 461.1702[M-CA]^−^	C_29_H_36_O_15_	Forsythoside A	8.53
13	59.379	446	445.0806[M-H]^−^, 269.0457[M-H-GlcA]^−^	C_21_H_18_O_11_	Baicalin	−6.73
14	72.113	460	459.0806[M-H]^−^, 283.0615[M-H-GlcA]^−^, 268.0380[283-15]^−^	C_22_H_20_O_11_	Oroxylin A-7-O-glucuronoside [28]	−4.41
15	74.464	460	459.0979[M-H]^−^, 283.0624[M-H-GlcA]^−^, 268.0381[283-15]^−^	C_22_H_20_O_11_	Wogonoside	8.21
16	90.078	284	283.0621[M-H]^−^, 268.0381[M-H-CH_3_]^−^	C_16_H_12_O_5_	Wogonin	−1.68
17	95.124		Unknown			
18	95.952	822	821.3988[M-H]^−^	C_42_H_62_O_16_	Glycyrrhizic acid	

**Table 3:** The contents of six markers in 16 batches of samples (*n* = 2).

No.	Contents (mg·g^−1^)					
	Geniposide	Paeoniflorin	Forsythoside	Baicalin	Forsythin	Wogonoside
S1	4.78	3.42	1.94	7.75	0.71	2.08
S2	4.96	3.04	6.06	10.13	0.94	2.64
S3	4.98	3.98	4.85	10.54	0.91	2.52
S4	5.31	3.26	4.46	10.45	1.02	2.53
S5	5.02	3.49	4.82	10.39	1.12	2.79
S6	4.96	3.04	6.06	10.13	0.94	2.64
S7	4.76	2.83	6.75	8.38	1.07	2.08
S8	4.78	3.79	5.09	9.29	1.03	2.28
S9	4.60	3.03	2.79	11.11	0.95	2.80
S10	4.49	2.55	3.37	8.35	0.84	2.24
S11	5.08	3.30	5.00	11.37	0.93	2.80
S12	5.42	3.24	3.82	10.97	0.57	2.83
S13	4.93	2.96	6.18	9.97	1.15	2.50
S14	5.12	3.04	6.72	10.28	1.27	2.57
S15	5.16	2.82	6.02	9.53	1.06	2.54
S16	4.91	2.94	6.23	10.29	1.08	2.65

## Data Availability

The data used to support the findings of this study are included within the article.

## Supplementary Materials

Supplementary Materials Figure S1: structures of the common markers from the HPLC fingerprint of XCQG by HPLC-Q/TOF-MS.
