# Supplementary material for: A Preferable Approach for the Quality Control of Xiaoer Chiqiao Qingre Granules Based on the Combination of Chromatographic Fingerprints and Chemometrics
Source: J Anal Methods Chem. 2020 Sep 29;2020:6836981. doi: 10.1155/2020/6836981 (PMC7542504; doi:10.1155/2020/6836981)
Supplement: Supplementary Materials — Figure S1: structures of the common markers from the HPLC fingerprint of XCQG by HPLC-Q/TOF-MS. [file 6836981.f1.pdf]

Supplementary Information

A preferable approach for quality control of Xiaoer Chiqiao Qingre Granules based on the combination of chromatographic fingerprints and chemometrics

Tong Xu[1,3#], Xiaoqi Li[1,2 #], Mengmeng Huang[1,2], Qi Wang[1,2], Chao Li[4], Gang Tian[4], Yan Chen[1,2]

[1]Affiliated Hospital of Integrated Traditional Chinese and Western Medicine, Nanjing University of Chinese Medicine, Nanjing 210028, China

[2]Multi-component of traditional Chinese medicine and Microecology Research Center, Jiangsu Province Academy of traditional Chinese medicine, Nanjing 210028, China

[3]The State Key Laboratory of Natural Medicines, China Pharmaceutical University, Nanjing 210009, China

[4]Jumpcan Pharmaceutical Co., Ltd, Taixing 225400, China

Correspondence

Prof. Dr. Yan Chen, Affiliated Hospital of Integrated Traditional Chinese and Western Medicine, Nanjing University of Chinese Medicine, Shizi Street 100, Hongshan Road, Nanjing,China, E-mail: ychen202@hotmail.com, Phone.: +86 25 52362155

Co-first authors

[#]These two authors, Tong Xu and Xiaoqi Li, contributed to the work eqully and should be regarded as co-first authors.

Table of contents

1. The theory of multi-wavelength fusion fingerprint method

2. Structures of the common markers from the HPLC fingerprint of XCQG by HPLC-Q/TOF-MS (Fig. S1)

1. Theory of multi-wavelength fusion fingerprint method

A multi-wavelength fusion fingerprint was established by principal component analysis (PCA) strategy. The AIA format data of 230 nm, 250 nm and 330 nm were respectively exported from the Agilent ChemStation chromatographic workstation (C03.01), and then put into the "Similarity Evaluation System for Chromatographic Fingerprint of TCM"(Version 2004 A) to get the text files. Prescribe DAD measurement data as matrix X (n×m), detection wavelengths as rows (n), and retention times as columns (m). X can be described as m single-wavelength at various wavelengths, and each fingerprint is measured at n different time, as shown as the following equation.

$$X=[x_1, x_2, x_3,\ldots, x_m] \quad (1)$$

Then the matrix was decomposed complied by the principal component analysis strategy.

$$X = TV^T+E \quad (2)$$

Where X is the matrix, T is the score matrix, V is the loading matrix and E is the residue matrix. After ignoring the residues, the score matrix can be expressed as the following equation:

$$T= XV \quad (3)$$

Since the first principal component can explain the variables as many as possible, it is chosen to describe the entire matrix.

$$t_1=XV_1= v_{11}x_1 + v_{21}x_2+\ldots\ldots+ v_{m1}x_m \quad (4)$$

Where x_m is the chromatographic fingerprint at the *m*th wavelength andv_{m1} is a

constant. It is found that the first column of the matrix could be described as a linear combination of each chromatographic fingerprint. While after normalizing the peak area, we could ultimately get the multi-wavelength fusion fingerprint.

2. Structures of the common markers from the HPLC fingerprint of XCQG by HPLC-Q/TOF-MS (Fig. S1)

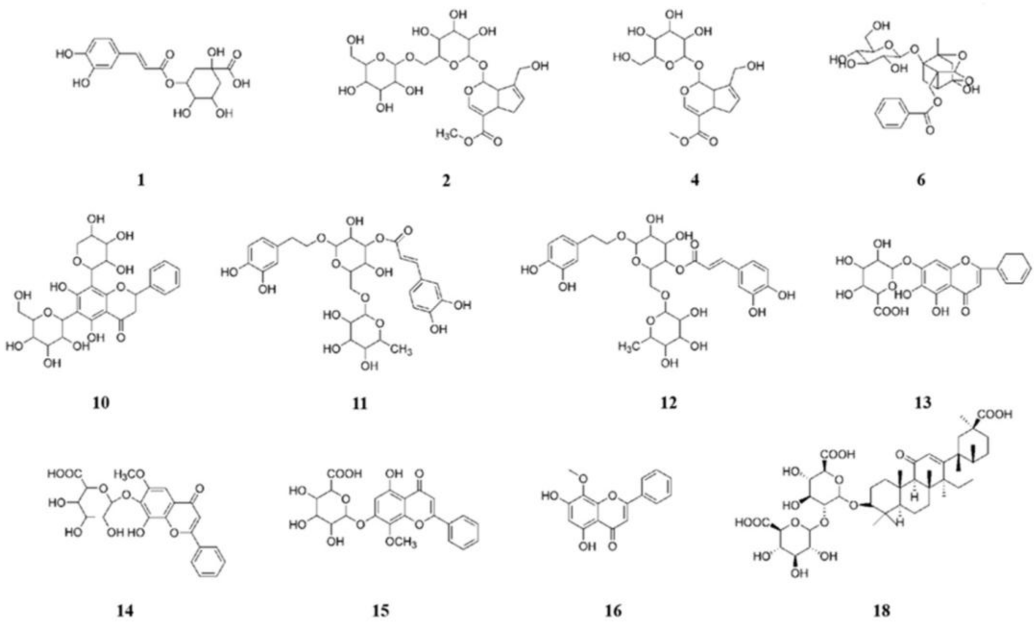

Fig. S1 Structures of the common markers from the HPLC fingerprint of XCQG by HPLC-Q/TOF-MS
